# The Short Isoform of DNAJB6 Protects against 1-Methyl-4-phenylpridinium Ion-Induced Apoptosis in LN18 Cells via Inhibiting Both ROS Formation and Mitochondrial Membrane Potential Loss

**DOI:** 10.1155/2017/7982389

**Published:** 2017-02-09

**Authors:** Yeon-Mi Hong, Yohan Hong, Yeong-Gon Choi, Sujung Yeo, Soo Hee Jin, Sae-Won Lee, Backil Sung, Sook-Hyun Lee, Hyejin Jung, Sabina Lim

**Affiliations:** ^1^Research Group of Pain and Neuroscience, East-West Medical Research Institute, Kyung Hee University, Seoul, Republic of Korea; ^2^Department of Meridian & Acupoint, College of Korean Medicine, Kyung Hee University, Seoul, Republic of Korea; ^3^Department of Neurodegenerative Diseases, Ilsong Institute of Life Science, Hallym University, Anyang, Republic of Korea; ^4^Department of Meridian & Acupoint, College of Korean Medicine, Sangji University, Wonju, Republic of Korea; ^5^Biomedical Research Institute and IRICT, Seoul National University Hospital, Seoul, Republic of Korea; ^6^Department of Biology, Pacific Union College, Angwin, CA 94508, USA

## Abstract

In a previous study, we found that the short isoform of DNAJB6 (DNAJB6(S)) had been decreased in the striatum of a mouse model of Parkinson's disease (PD) induced by 1-methyl-4-phenyl-1,2,3,6-tetrahydropyridine (MPTP). DNAJB6, one of the heat shock proteins, has been implicated in the pathogenesis of PD. In this study, we explored the cytoprotective effect of DNAJB6(S) against 1-methyl-4-phenylpyridinium ion- (MPP^+^-) induced apoptosis and the underlying molecular mechanisms in cultured LN18 cells from astrocytic tumors. We observed that MPP^+^ significantly reduced the cell viability and induced apoptosis in LN18 glioblastoma cells. DNAJB6(S) protected LN18 cells against MPP^+^-induced apoptosis not only by suppressing Bax cleavage but also by inhibiting a series of apoptotic events including loss of mitochondrial membrane potential, increase in intracellular reactive oxygen species, and activation of caspase-9. These observations suggest that the cytoprotective effects of DNAJB6(S) may be mediated, at least in part, by the mitochondrial pathway of apoptosis.

## 1. Introduction

Heat shock proteins (HSPs) are molecular chaperones that were first described in relation to their role in the response to heat shock [1]. An important function of HSPs is to protect against a variety of adverse conditions by refolding misfolded proteins and accelerating the degradation of aggregates of these proteins [2, 3]. DNAJB6, a member of the heat shock protein 40 (HSP40) family, a noncanonical member of the DNAJ-chaperone family, plays various roles in mammalian development, recovery from misfolded protein aggregates, and self-renewal of nervous cells [4]. DNAJB6 exists as two spliced isoforms characterized by alternative C-termini. Full-length DNAJB6(L) (38 kDa) predominantly exhibits nuclear localization due to the presence of a C-terminal nuclear localization sequence, whereas the short isoform DNAJB6(S) (27 kDa) lacks the localization signal and is therefore predominantly cytoplasmically located [5, 6]. DNAJB6(L) isoform is not effective with suppressing cytoplasmic protein aggregation while DNAJB6(S) isoform is suppressing protein aggregation effectively in the cytoplasm [7]. DNAJB6 is highly upregulated in Parkinsonian astrocytes, which might reflect a protective reaction [8].

The mitochondrial toxin 1-methyl-4-phenylpyridinium ion (MPP^+^), an inhibitor of complex I, increases mitochondria-dependent reactive oxygen species (ROS) generation and induces caspase-dependent apoptotic cell death in the mitochondria [9–12]. MPP^+^ causes permanent symptoms of PD by destroying dopaminergic (DA) neurons in the substantia nigra and has been widely used to reproduce biochemical alterations linked to PD in vitro [13–15]. MPP^+^ is produced in the astrocytes of the brain and is taken into DA neurons by dopamine transporters [16, 17]. Interestingly, DNAJB6(S) expression decreases in the striatum of mice after administration of 1-methyl-4-phenyl-1,2,3,6-tetrahydropyridine (MPTP), a prodrug of MPP^+^ [18]. However, the role of DNAJB6(S) in DA neuron degeneration remains unclear.

Based on our previous research [18], we hypothesized that DNAJB6(S) would protect cells against MPP^+^-induced apoptosis. Intrinsic and extrinsic pathways of apoptosis are well characterized in mammalian cells [19, 20]. The intrinsic pathways of apoptosis are initiated by a mitochondria-dependent process that induces release of cytochrome c, activation of caspase-9 and -3, and consequent cell death [21]. The Bcl-2 family of proteins is critical for the regulation of apoptosis in many varieties of cells, and its members are categorized by specific function as antiapoptotic (e.g., Bcl-2, Bcl-XL) and proapoptotic (e.g., Bad, Bax, and Bid) [22]. Proapoptotic Bax is essential for initiating the intrinsic pathways of apoptosis [23] and translocates from the cytosol to the mitochondria during apoptosis [24]. Therefore, to gain more mechanistic insight into the role of DNAJB6(S), we explored the cytoprotection of DNAJB6(S) against MPP^+^-induced apoptosis and the molecular mechanisms underlying this process in cultured LN18 cells from astrocytic tumors.

## 2. Materials and Methods

### 2.1. Materials

The human glioblastoma cell lines A172 (#CRL-1620), LN18 (#CRL-2610), U87MG (#HTB-14), and LN229 (#CRL-2611) were purchased from the American Type Culture Collection (ATCC, Manassas, VA). Human DNAJB6(S) cDNA (DnaJ/HSP40 homologue, subfamily B, member 6, NM_005494, small variant 241-aa, #SC110111) was purchased from OriGene Technologies Inc. (Rockville, MD). DNAJB6(S) siRNAs (Cat. number 1042869 duplex) and a negative control siRNA (Cat. number SN-1002) were purchased from Bioneer (Seoul, Korea). DMEM, pCR®2.1-TOPO vector®, vector pcDNATM3.1/myc-His (−) type A, Lipofectamine™ LTX and PLUS Reagent, G418 sulfate, Lipofectamine RNAiMAX, CM-H2DCFDA, 5,5′,6,6′-tetrachloro-1,1′,3,3′-tetraethylbenzimidazolylcarbocyanine iodide (JC-1), SDS-PAGE (4–12% Tris-Bis mini gels), and anti-c-myc antibody are all from Invitrogen (Carlsbad, CA). FBS and Mitochondrial Isolation kit are from Thermo Scientific (Waltham, MA), glutamine and penicillin/streptomycin are from Welgene (Kyungsan, Korea), MPP^+^ and *β*-actin antibody are bought from Sigma-Aldrich (St. Louis, MO, USA), restriction enzymes* Bam*HI and* Hin*dIII (NEB) and RNeasy Mini Spin Columns are from Qiagen (Hiden, Germany), Transcriptor First-Strand cDNA Synthesis kit is from Roche Diagnostics (Basel, Switzerland), anti-DNAJB6 antibody (1 : 1000) is from Abnova (Taipei, Taiwan, H00010049-M01), anti-c-myc antibody (1 : 3000) is from Invitrogen (R950-25), anti-Bax antibody (1 : 1,000) is from Cell Signaling (2772), anti-COX-2 antibody (1 : 2000) is from Cell Signaling (4842), and anticleaved caspase-9 antibody (1 : 1,000) is from Cell Signaling (9509). MitoTracker® Red CMXRos is from Molecular Probe (Eugene, OR), Annexin V-FITC apoptosis detection kit is form BD Pharmingen Inc. (San Diego, CA), and RIPA buffer is from Thermo Scientific (Waltham, MA).

### 2.2. Cell Cultures

We chose LN18 cell line, one of the glioblastoma cell lines, because astrocytes (glial cells) in the patient tissue samples expressed DNAJB6 protein [4] and because DNAJB6 gene was expressed in the striatum by MPTP treatment [18]. To identify a cell line that expresses high levels of DNAJB6 among human glioblastoma cell lines from astrocytic tumors, A172, LN18, U87MG, and LN229 cell lines were cultured in a humidified 5% CO_2_ environment at 37°C in Dulbecco's modified Eagle's medium with 4.5 g/L glucose containing heat-inactivated FBS (5% for LN18 and LN129, 10% for A172 and U87MG), 2 mM L-glutamine, 50 U/mL penicillin, and 50 *μ*g/mL streptomycin. We examined the endogenous protein level of DNAJB6(S) in various human glioblastoma cell types and found that DNAJB6(S) was constitutively expressed in LN18 and U87MG cells, while other cell lines including A172 and LN229 did not express observable amounts of DNAJB6. Therefore, we selected LN18 cells for the current study (Supplementary Figure 1 in Supplementary Material available online at https://doi.org/10.1155/2017/7982389).

### 2.3. Plasmid Construction

To construct the mammalian expression plasmid, the DNAJB6(S) fragment was subcloned by PCR into the pCR 2.1-TOPO vector using the forward primer 5′-GTGGATCCATGGTGGATTACTATG-3′ and the reverse primer 5′-CCAAGCTTCTTGTTATCCAAGCG-3′, which contained restriction sites for* Bam*HI and* Hin*dIII, respectively. The cycling conditions consisted of denaturation at 94°C for 2 min; followed by 30 cycles of 94°C for 1 min, 59°C for 1 min, and 72°C for 1 min 30 sec; and final annealing/extension at 72°C for 10 min. The PCR product was analyzed by 1% agarose gel electrophoresis, and the identity of the amplified DNA was confirmed by sequencing analysis (Cosmo Gentech, Seoul, Korea). The PCR product was then digested with the* Bam*HI and* Hin*dIII enzymes and ligated into the vector pcDNATM3.1/myc-His (−) type A for transfection into LN18 glioblastoma cells.

### 2.4. Generation of Stable Cell Lines

LN18 cells were transfected with 10 *µ*g of the recombinant plasmid (pcDNA™3.1/*myc*-His/DNAJB6(S)) or the vector alone (pcDNA3.1/*myc*-His) for 48 h using Lipofectamine LTX and PLUS Reagent according to the protocol provided by Invitrogen, followed by screening with 800 *μ*g/mL G418 sulfate. Colonies derived from a single cell were selected and expanded as stable cell lines. These clones were screened for DNAJB6(S) protein expression by western blot analysis.

### 2.5. Total RNA Isolation and Reverse-Transcription PCR

Frozen cells were thawed on ice and homogenized with RNase-free tissue grinders in 200 *µ*L of Trizol. RNA was purified using RNeasy Mini Spin Columns according to the manufacturer's protocol. Total RNA (2 *µ*g) was reverse transcribed using a Transcriptor First-Strand cDNA Synthesis kit. DNAJB6(S) was amplified by PCR as follows: denaturation at 92°C for 2 min; followed by 30 cycles of 1 min at 94°C, 1 min at 56°C, and 1 min at 70°C; and final annealing/extension of 10 min at 70°C, using the same primers used for plasmid construction. The reference gene glyceraldehyde-3-phosphate dehydrogenase (GAPDH) was amplified by PCR as follows: denaturation at 92°C for 2 min; followed by 25 cycles of 1 min at 94°C, 1 min at 55°C, and 1 min at 72°C; and final annealing/extension of 10 min at 70°C, using the forward primer 5′-GTCTTCACCATGGAGAAGG-3′ and the reverse primer 5′-TCATGGATGACCTTGGCCAG-3′. The PCR products were resolved on 1.5% agarose gel, stained with ethidium bromide, and visualized with UV light.

### 2.6. siRNA Transfection

We examined the level of DNAJB6(S) in cells transiently overexpressing different siRNA. Three independent DNAJB6(S) siRNAs and a negative control siRNA were used. LN18 cells were cotransfected with 1 *µ*g pDNAJB6(S) and 3 *µ*g siRNA per well (in a 6-well culture plate) using Lipofectamine RNAiMAX according to the manufacturer's protocol. Medium was changed 24 h after transfection, and cells were used for immunoblotting or RT-PCR analysis 48 h after transfection.

### 2.7. Immunocytochemistry

Approximately 1 × 10^5^ cells/well were cultured on coverslips for 48 h and then treated with or without 500 *µ*M MPP^+^ for 48 h. For immunohistochemistry, cells were fixed with 4% paraformaldehyde for 10 min and permeabilized with 0.05% Triton X-100 for 5 min. The cells were blocked with 5% horse serum for 1 h and stained with anti-DNAJB6 antibody (1 : 300) overnight at 4°C, followed by a FITC-conjugated goat anti-mouse secondary antibody (1 : 1000) for 1 h. Confocal images were obtained with a Zeiss confocal microscope (Oberkochen, Germany) at excitation/emission wavelengths of 488/510 nm to visualize FITC. For mitochondrial staining, living cells were washed with phosphate-buffered saline (PBS) and stained with MitoTracker Red CMXRos (1 : 5,000) for 10 min then fixed with 4% paraformaldehyde for 10 min. Following several washes, the mitochondria were observed and photographed with an Olympus fluorescence microscope (Olympus BX51, Tokyo, Japan).

### 2.8. Flow Cytometric Analysis of Apoptosis

Apoptosis was analyzed using an Annexin V-FITC apoptosis detection kit according to the protocol provided by BD Pharmingen Inc. Approximately 1 × 10^5^ cells/well were cultured in 6-well plates and each well was treated with DMEM/1% FBS with or without MPP^+^ for 24 h or 48 h. The cells were collected by centrifugation and washed twice with cold PBS. The pellets were resuspended in 100 *µ*L 1x binding buffer and 5 *µ*L propidium iodide (PI); 5 *µ*L of Annexin V was then added. After incubation for 15 min in the dark at room temperature, 400 *µ*L of 1x binding buffer was added to the mixture. Apoptosis was then analyzed by flow cytometry (BD FACSCalibur; BD Biosciences, San Jose, CA). Quadrant positioning was performed using Annexin V/PI dot plots. The numbers of early (Annexin V^+^/PI^−^) and late (Annexin V^+^/PI^+^) apoptotic cells are expressed as percentages [25].

### 2.9. Flow Cytometric Analysis of Oxidative Stress

The level of intracellular ROS was evaluated using the fluorescent probe chloromethyl-H2-2′,7′-dichlorofluorescein diacetate (CM-H2DCFDA) according to the manufacturer's protocol. Cells were collected by trypsinization, washed with PBS, and incubated with 2 *µ*M CM-H2DCFDA in 0.5 mL of PBS for 15 min at room temperature. The cells were washed twice with PBS, and the pellets were resuspended in 0.5 mL of PBS. The fluorescence intensity at 530 nm (FL-1) of 10,000 cells was measured using flow cytometry.

### 2.10. Mitochondrial Membrane Potential (Δ*ψ*_*m*_) Assay Using Flow Cytometry

Δ*ψ*_*m*_ was analyzed using the cationic dye JC-1 according to the manufacturer's protocol. A JC-1 stock solution was prepared at 1 mg/mL in dimethyl sulfoxide (DMSO) and stored at −20°C until use. After MPP^+^ treatment, cells were collected by centrifugation and washed twice with cold PBS. The pellets were resuspended in 0.5 mL of PBS and incubated with 1 *µ*M JC-1 for 30 min at room temperature, shielded from light. The fluorescence intensities at 530 nm (FL-1) and 590 nm (FL-2) of 10,000 cells were measured using a flow cytometer. Δ*ψ*_*m*_ was calculated as the ratio of red (FL-2) to green (FL-1) fluorescence.

### 2.11. Mitochondrial Fraction Isolation

Mitochondrial protein extraction was performed using the Mitochondrial Isolation kit according to the manufacturer's protocol. Briefly, Reagent A was added to collected cells, which were then incubated for 2 min on ice. Cells were homogenized with ice-cold buffer A for approximately 30 strokes in a Dounce homogenizer. Reagent C was added to the lysate, and it was centrifuged at 700 ×g for 10 min at 4°C. Following centrifugation, the supernatant was gathered and centrifuged at 12,000 rpm for 15 min at 4°C. The supernatant was saved as the cytosolic fraction and the pellet (mitochondrial fraction) was washed in Reagent C and lysed with 2% CHAPS in Tris-buffered saline. The supernatant of the both fractions was concentrated by a centrivap concentrator (Labconco, Kansas City, MO, USA) and the protein concentration was determined using bicinchoninic acid (BCA). A sample of 10 *µ*g of total protein was used for the western blot.

### 2.12. Western Blot Analysis

For western blot analysis, cells were homogenized in radioimmunoprecipitation assay buffer (RIPA) on ice for 30 min. The lysate was centrifuged at 12,000 rpm at 4°C for 20 min, and the protein concentration of the supernatant was determined using BCA. A sample of 30 *µ*g of total protein was separated by SDS-PAGE (4–12% Tris-Bis mini gels) and transferred to PVDF membranes. The membranes were blocked with 5% skim milk with 0.05% Tween 20 in Tris-buffered saline at 37°C for 1 h and incubated overnight at 4°C with mouse anti-DNAJB6 (1 : 1,000), mouse anti-c-myc (1 : 3000), rabbit anti-Bax (1 : 1,000), and rabbit anticleaved caspase-9 (1 : 1,000) antibodies. The membranes were then incubated with horseradish peroxidase- (HRP-) conjugated anti-mouse or anti-rabbit IgG antibodies (1 : 5,000) for 1 h and visualized using a chemiluminescent substrate. The band densities were determined using ImageJ (https://rsbweb.nih.gov/ij/) and normalized against those detected by an anti-*β*-actin antibody (1 : 5,000).

### 2.13. Statistical Analysis

Unless otherwise specified, data were analyzed using one-way analysis of variance (ANOVA) followed by Duncan multiple range test (DMRT) at *p* = 0.05. AII statistical analyses were performed with SPSS version 23.0 software package for Windows (SPSS Inc., Chicago, IL).

## 3. Results 

### 3.1. Identification of the DNAJB6(S) Gene

**Figure 1:**
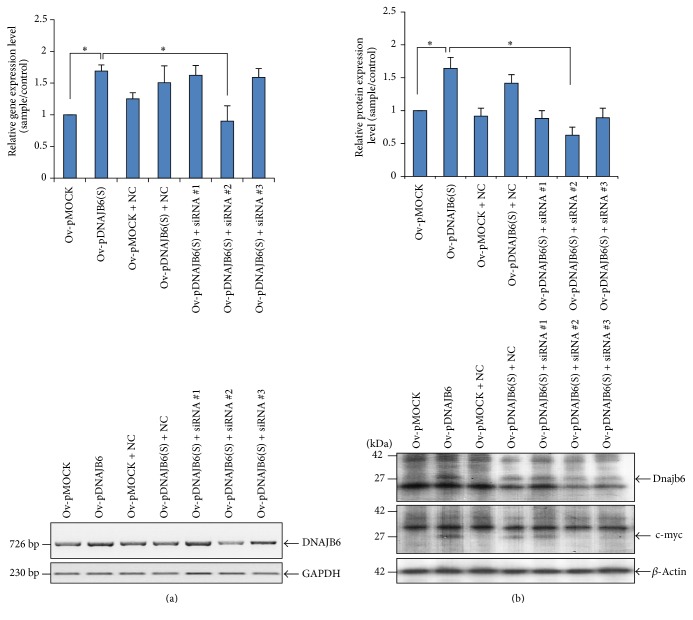
Transient overexpression of the recombinant plasmid DNAJB6(S) and small interfering RNAs in LN18 glioblastoma cells. LN18 cells were transiently transfected to overexpress (Ov) pMOCK or DNAJB6(S) alone or were cotransfected with each of the vectors together with negative control siRNA (NC) or three different DNAJB6(S) siRNAs (siRNA 1, siRNA 2, or siRNA 3). Cells were analyzed to determine mRNA levels (a) and immunoblotted with an anti-DNAJB6 antibody or c-myc epitope-tagged antibody (b). Data are expressed relative to pMOCK cells and presented as means ± SEM (*N* = 3, ^*∗*^*p* < 0.05). GAPDH and *β*-actin levels are shown as internal controls.

The short isoform DNAJB6(S) over the long isoform DNAJB6(L) is chosen for this study, that is, to investigate whether or not DNAJB6 protein is involved in the apoptotic control of cells because DNAJB6(S) is predominantly located in the cytoplasm where apoptosis occurs while DNAJB6(L) is translocated to the nucleus using localization sequence in the C-terminal [5, 6]. LN18 cells were transiently transfected to overexpress (Ov) a mock plasmid (pMOCK) or DNAJB6(S). To identity the* DNAJB6(S)* gene, cells were cotransfected with DNAJB6(S) and one of three different DNAJB6(S) siRNAs (siRNA 1, 2, or 3). DNAJB6(S) level was determined by RT-PCR and western blot. Transfections with siRNA 2 significantly silenced DNAJB6(S) mRNA (Figure 1(a)) and protein expression (*p* < 0.05) as well as the c-myc epitope-tagged protein in DNAJB6(S) (Figure 1(b)). The expression level of protein in DNAJB6(S) cells was 2-fold higher than that in pMOCK cells (*p* < 0.05) and the effect of siRNA-mediated c-myc silencing in cells cotransfected with pDNAJB6(S) and siRNA 2 was almost the same as for the DNAJB6(S) protein (Figure 1(b), low panel). These data show that the recombinant plasmid DNAJB6(S) was successfully transfected and that the transcript and protein were expressed in LN18 cells.

### 3.2. MPP^+^ Induces Apoptotic Cell Death in LN18 Cells

To determine the effects of MPP^+^ on apoptosis, LN18 cells were exposed to 0 (Ctrl), 300, or 500 *µ*M MPP^+^ for 24 or 48 h, and apoptosis was analyzed using flow cytometry. The percentages of Annexin V-stained populations with or without PI staining (V^+^/PI^−^: early apoptotic cells, V^+^/PI^+^: late apoptotic cells) are presented in Supplementary Figure 2(a). LN18 cells exposed to 500 *µ*M MPP^+^ for 48 h showed significantly more apoptosis (26.48%) than the control cells (10.21%) (*p* < 0.05). At 48 h of 500 *µ*M treatment, cell bodies appeared to swell and lose their normal shape, tended to disintegrate, and were clearly vacuolated (Supplementary Figures 2(f) and 2(g)). Therefore, culture conditions of 48 h of incubation and a concentration of 500 *μ*M MPP^+^ were applied hereafter in this study. We also assessed the changes in expression level of DNAJB6(S) after MPP^+^ treatment by western blotting. After 48 h of MPP^+^ treatment at the concentration of 500 *µ*M, the endogenous level of DNAJB6(S) in LN18 cells decreased by approximately 30% compared to the untreated cells (Supplementary Figure 2(h)).

### 3.3. Transient Overexpression of DNAJB6(S) Reduces MPP^+^-Induced Apoptosis

**Figure 2:**
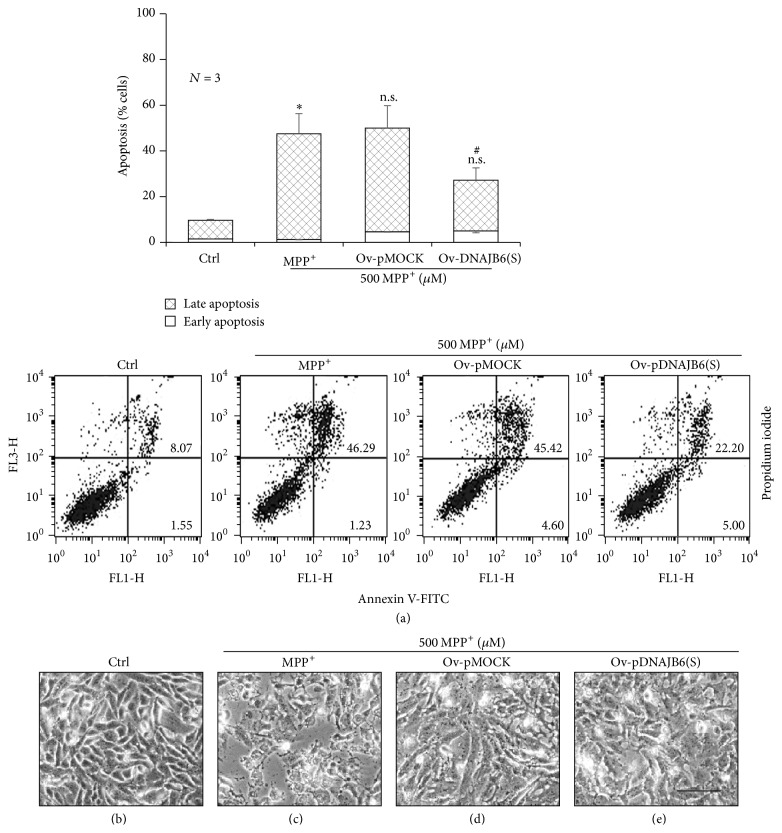
DNAJB6(S) transient overexpression protects against MPP^+^-induced apoptosis. (a) LN18 cells transiently overexpressing a control plasmid (pMOCK) or pDNAJB6(S) were exposed to 500 *µ*M MPP^+^ for 48 h, and apoptosis was analyzed using flow cytometry. The total numbers of early (Annexin V^+^/PI^−^) and late (Annexin V^+^/PI^+^) apoptotic cells are expressed as percentages. (*N* = 3, mean ± SEM, ^*∗*^*p* < 0.05 and ^n.s.^*p* > 0.05 compared with control at 48 h, and ^#^*p* < 0.05 compared with Ov-pMOCK.) (b) Cell morphological changes and patterns were visualized using a phase-contrast microscope. Bar is 100 *µ*m.

After transient transfection with a DNAJB6(s) overexpression plasmid (Ob-pDNAJB6(s)) or mock transfection (Ov-pMOCK) for 24 h, cells were exposed to 500 *µ*M MPP^+^ for up to 48 h and apoptosis was analyzed by flow cytometry. Untransfected cells acted as control, and the transfected cells were treated with MPP^+^ (MPP^+^-treated) or vehicle only (control). Apoptosis was significantly increased in MPP^+^-treated and Ov-pMOCK cells compared to the control cells (Figure 2) (*p* < 0.05) and reduced in Ov-pDNAJB6(S) cells compared to MPP^+^-treated and Ov-pMOCK cells (*p* < 0.05) (Figure 2(a)). Consistent with these results, morphological observations at 48 h revealed that MPP^+^-treated cells had lost their normal shape and looked unhealthy (Figure 2(c)) compared with the control (Figure 2(b)).

### 3.4. Establishment of DNAJB6(S) Stably Overexpressing LN18 Cells

**Figure 3:**
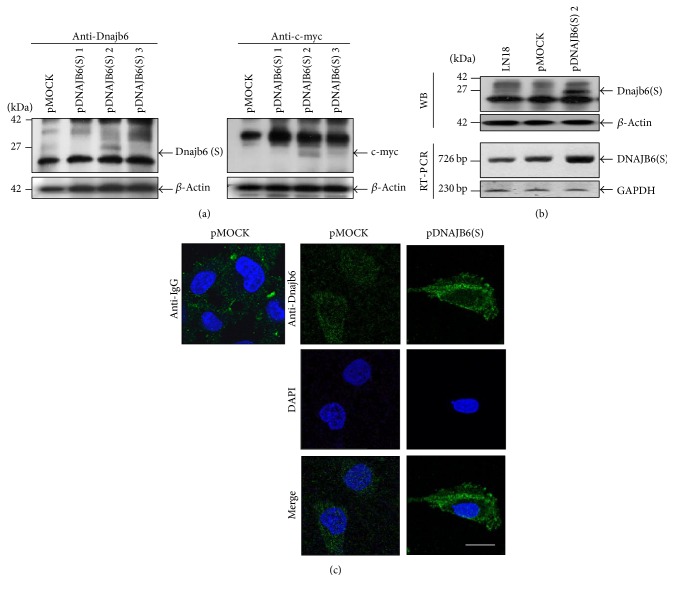
Establishment of DNAJB6(S) stable overexpressing LN18 cells. (a) The protein levels of DNAJB6(S) (left) and the protein levels of recombinant DNAJB6(S) with a C-terminal c-myc epitope (right) in LN18 cell lines stably transfected by pMOCK or pDNAJB6(S) were determined by western blotting using an anti-DNAJB6 or anti-c-myc antibody. (b) The expression levels of DNAJB6(S) in pMOCK cells and pDNAJB6(S)-2 cells were determined by western blot analysis and RT-PCR. (c) Subcellular localization of DNAJB6(S). Staining with anti-mouse IgG was performed as a negative control (left), and FITC secondary antibody was used to detect specific immunostaining; nuclei were stained with DAPI (middle panel). Bar is 10 *µ*m, 1000x magnification.

To investigate how DNAJB6(S) is regulated in response to MPP^+^ and reveal the precise cell survival pathway involved, we generated LN18 cells line stably overexpressing DNAJB6(S) (pDNAJB6(S) cells). Western blotting, RT-PCR, and immunocytochemistry were used to evaluate the maximum level of DNAJB6(S) protein in the cells. In the western blot analysis, an anti-DNAJB6 monoclonal antibody was used to recognize the DNAJB6(S) protein, and an anti-c-myc epitope antibody detected the myc tag present at the C-terminus of the recombinant DNAJB6(S) protein as well as the myc tag alone (Figure 3(a)). The protein and mRNA expression levels of DNAJB6(S) in cell line number 2 were 3 and 4 times higher, respectively, than those in LN18 cells transfected with empty vector pcDNA3.1 (pMOCK cells) (Figure 3(b)). Cell line number 2 showed a predominantly cytoplasmic distribution under resting conditions, with a twofold increase in expression compared with pMOCK cells (Figure 3(c)).

### 3.5. Stable Overexpression of DNAJB6(S) Attenuates MPP^+^-Induced Apoptosis

**Figure 4:**
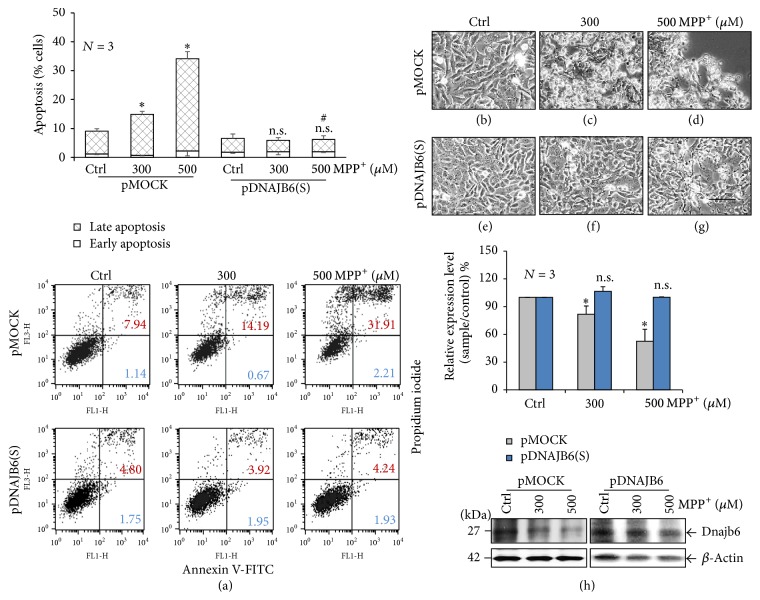
DNAJB6(S) stable overexpression in LN18 cells protects against apoptosis. (a) LN18 cells stably transfected by pMOCK or pDNAJB6(S) were exposed to either vehicle (Ctrl) or 500 *µ*M MPP^+^ for 48 h, and apoptosis was assessed using flow cytometry. The total numbers of early (Annexin V^+^/PI^−^) and late (Annexin V^+^/PI^+^) apoptotic cells are expressed as percentages. (b)–(g) Cell morphological changes and patterns were visualized. Bar is 100 *µ*m. (h) Protein levels of DNAJB6(S) were evaluated by western blot assay after treatment with 500 *µ*M MPP^+^ for 48 h. *β*-Actin was used as an internal control. (*N* = 3, mean ± SEM, ^*∗*^*p* < 0.05 and ^n.s.^*p* > 0.05 compared with control-treated pMOCK or pDNAJB6 and ^#^*p* < 0.05 compared with MPP^+^-treated pMOCK).

To assess the effects of MPP^+^ treatment on the pDNAJB6(S) cells, we examined apoptosis at 48 h of treatment; the pMOCK and pDNAJB6(S) cells were exposed to either 300 or 500 *µ*M MPP^+^ (pMOCK-300, pMOCK-500, pDNAJB6(S)-300, and pDNAJB6(S)-500) and analyzed by flow cytometry. Both the pMOCK-300 and pMOCK-500 conditions significantly induced apoptosis compared to the pMOCK-control (*p* < 0.05). In contrast, both the pDNAJB6(S)-300 and the pDNAJB6(S)-500 conditions showed no significant induction of apoptosis compared to the pDNAJB6(S)-control (Figure 4(a)). The highest levels of apoptosis occurred in pMOCK-500 cells at 48 h. The pMOCK-300 and the pMOCK-500 cells showed a loss of viability (Figures 4(c) and 4(d)), whereas the pDNAJB6(S)-300 and pDNAJB6(S)-500 cells appeared to be healthy (Figures 4(f) and 4(g)). The pMOCK-500 cells significantly decreased compared to pMOCK-control cells by western blotting (52.3%, *p* < 0.05), whereas pDNAJB6(S)-500 cells did not change compared to pDNAJB6-control cells (Figure 4(h)).

### 3.6. DNAJB6(S) Attenuates the Formation of ROS in MPP^+^-Treated Cells

**Figure 5:**
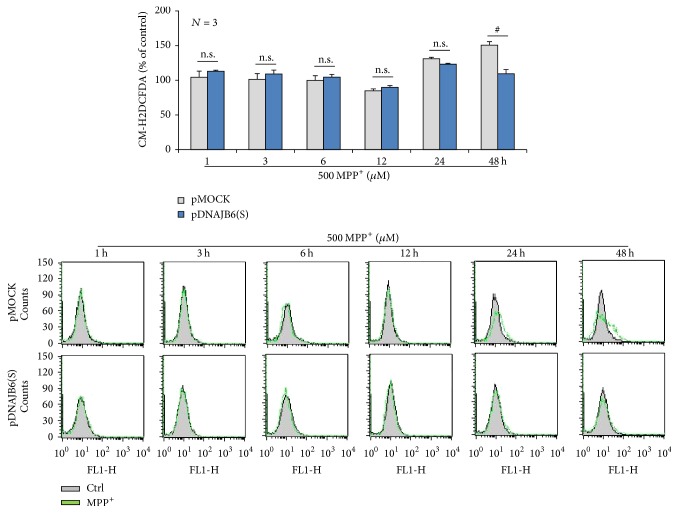
DNAJB6(S) attenuates the formation of ROS in MPP^+^-treated cells. LN18 cells stably transfected by pMOCK and pDNAJB6(S) were exposed to either vehicle (Ctrl) or 500 *µ*M MPP^+^ for 48 h, and intracellular ROS levels were measured with a CM-H2DCFDA probe using flow cytometry. The data were analyzed using Student's *t*-test. (*N* = 3, mean ± SEM, ^#^*p* < 0.05 and ^n.s.^*p* > 0.05 compared with pMOCK.)

Using the marker CM-H2DCFDA, a colorless and nonfluorescent leuco dye derived from dihydroxyfluorene, we examined intracellular ROS levels to analyze how they were affected by DNAJB6(S) after treatment with 500 *µ*M MPP^+^ for 1, 3, 6, 12, 24, or 48 h. pDNAJB6(S) cells readily took up the dye and its acetate groups were cleaved by intracellular esterase; subsequent oxidation produced a fluorescent adduct. Compared to MPP^+^-untreated cells (Ctrl), ROS formation in pMOCK cells treated with MPP^+^ for 24 and 48 h significantly increased (*p* < 0.05); however, ROS formation did not increase in MPP^+^-treated pDNAJB6(S) cells at any time point (*p* > 0.05) (Figure 5).

### 3.7. DNAJB6(S) Prevents the Loss of Mitochondrial Membrane Potential (Δ*ψ*_*m*_) in MPP^+^-Treated Cells

**Figure 6:**
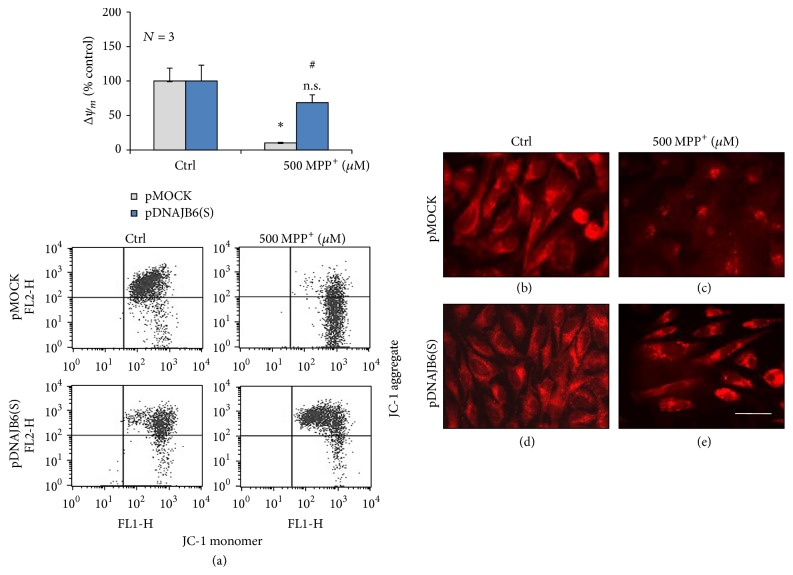
DNAJB6(S) prevents the loss of mitochondrial membrane potential (Δ*ψ*_*m*_) in MPP^+^-treated cells. (a) LN18 cells stably transfected by pMOCK and pDNAJB6(S) were exposed to either vehicle (Ctrl) or 500 *µ*M MPP^+^ for 48 h, and the loss in Δ*ψ*_*m*_ was measured by flow cytometry using the JC-1 mitochondrial probe. The values were calculated as the ratio of red/green signal and are expressed as percentage of the control. (*N* = 3, mean ± SEM, ^*∗*^*p* < 0.05 and ^n.s.^*p* > 0.05 compared with control-treated pMOCK or pDNAJB6 and ^#^*p* < 0.05 compared with MPP^+^-treated pMOCK). (b)–(e) Cells treated with the conditions described in (a) were fixed and stained with the mitochondrial marker MitoTracker Red CMXRos and visualized by fluorescence microscopy. Bar is 50 *µ*m.

Mitochondrial dysfunction has been shown to be involved in the induction of apoptosis and central to the apoptotic pathway. We examined Δ*ψ*_*m*_ in cells treated with or without 500 *µ*M MPP^+^ for 48 h using the marker JC-1, a dye that reversibly accumulates in mitochondrial membranes in a potential-dependent manner. Δ*ψ*_*m*_ in pMOCK-500 cells treated with 500 *µ*M MPP^+^ decreased by approximately 90% compared with pMOCK-control cells, whereas pDNAJB6(S)-500 cells treated with 500 *µ*M MPP^+^ showed a reduction of only 30% compared with pDNAJB6(S)-control cells (Figure 6(a)). Cells were fixed and stained with the mitochondrial marker MitoTracker Red CMXRos and then visualized by fluorescence microscopy. pMOCK-control (Figure 6(b)) and pDNAJB6(S)-control cells (Figure 6(d)) showed red fluorescent puncta throughout the cytoplasm, showing the typical grainy structure of mitochondria. Treatment with MPP^+^ led to a significant reduction in the number of mitochondria in pMOCK-500 cells (Figure 6(c)), showing the deposit of mitochondria only at the perinuclear region whereas these effects were attenuated in pDNAJB6(S)-500 cells (Figure 6(e)). These observations indicate that DNAJB6(S) is able to inhibit the mitochondrial damage induced by MPP^+^.

### 3.8. DNAJB6(S) Inhibits p18 Bax Cleavage and Caspase-9 Activation in MPP^+^-Treated Cells

**Figure 7:**
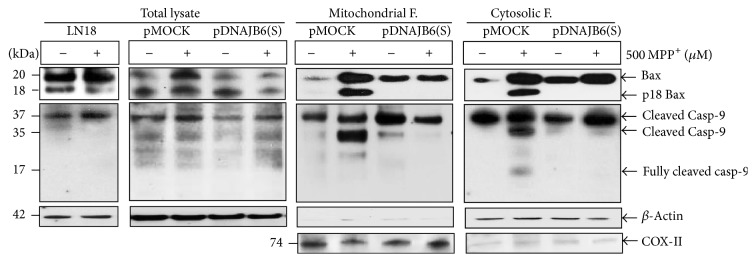
DNAJB6(S) inhibits p18 Bax cleavage and caspase-9 activation in MPP^+^-treated cells. LN18 cells stably transfected by pMOCK or pDNAJB6(S) were treated with or without 500 *µ*M MPP^+^ for 48 h. The cells were harvested, and mitochondrial and cytosolic fractions (F) were prepared. The expression levels of p18 Bax and caspase-9 cleavage were analyzed by western blotting. An anti-Bax antibody detected both full-length Bax (20 kDa) and p18 Bax. *β*-Actin was used as a loading control and COX-II was used for mitochondrial marker.

To explore the possible inhibitory effect of DNAJB6(S) on Bax activity, pDNAJB6(S) cells were treated with or without 500 *µ*M MPP^+^for 48 h, and the LN18 cell, total lysate, and mitochondrial and cytosolic fractions were isolated and analyzed by western blotting. Bax (20 kDa) was found in the total lysate of LN18, pMOCK, and pDNAJB6(S) as well as in both the mitochondrial and cytosolic fractions, regardless of MPP^+^ treatment, and constitutive expression of Bax was detected in all cell types (Figure 7, upper panels). However, MPP^+^-treated pMOCK cells showed a dramatic increase in Bax cleavage to its truncated/cleaved form of Bax (p18 Bax) in both the mitochondrial and cytosolic fractions when compared with untreated pMOCK cells; Bax cleavage was not observed in pDNAJB6(S) cells. These results indicate that overexpression of DNAJB6(S) inhibits the MPP^+^-induced formation of p18 Bax.

To explore the possible inhibitory effects of DNAJB6(S) on caspase activation following MPP^+^treatment, we examined the activation of caspase-9 by western blotting. The amount of the cleaved active form of caspase-9 (37 kDa) increased in the total lysate pMOCK cells treated with 500 MPP^+^ compared with cells untreated with 500 MPP^+^. Also, caspase-9 (37 kDa) slightly increased in the total lysate pDNAJB6(S) cells treated with MPP^+^ compared with cells untreated with 500 MPP^+^ (Figure 7, second middle panels).

The expression level of 37 kDa fragment of caspase-9 was detected in both the mitochondrial and cytosolic fraction, with or without MPP^+^ treatment (Figure 7, center panels). Upon treatment of pMOCK cells with MPP^+^, the 35 kDa fragment of caspase-9 was significantly increased in the cytosolic fraction (*p* < 0.05), whereas it increased only slightly in MPP^+^-treated pDNAJB6(S) cells, which indicates that DNAJB6(S) evidently inhibited the caspase-9 cleavage in the cytoplasm. Moreover, caspase-9 was cleaved to produce a 17 kDa proteolytic product in MPP^+^-treated pMOCK cells, but this band was not observed in pDNAJB6(S) cells. COX-II was used for mitochondrial marker.

## 4. Discussion

In this study, we demonstrated that DNAJB6(S)-overexpressing LN18 cells (pDNAJB6(S)) were significantly protected against apoptosis after exposure to 500 *µ*M MPP^+^ for 48 h (*p* < 0.05). The mitochondrial toxin 1-methyl-4-phenylpyridinium (MPP^+^), an inhibitor of complex I, has been widely used to reproduce biochemical alterations linked to PD in vitro [13–15]. MPP^+^ significantly reduced cell viability and induced apoptosis of LN18 cells in this study as well. Expression of DNAJB6(S) reduced both MPP^+^-induced Bax expression and cleavage to its p18 Bax form at 48 h. Bax is cleaved at aspartate 33 (Asp 33) by calpain into 18 kDa fragment (p18 Bax) during stress-induced apoptosis [26] and this p18 Bax appeared not only in the mitochondria membrane fraction during the caspase-dependent cell death but also in the cytosolic fraction during the caspase-independent cell death paradigm according to the type of stress applied [27]. We found Bax in the whole-cell lysate and in both the mitochondrial and cytosolic fractions, regardless of MPP^+^ treatment. However, MPP^+^-treated pMOCK cells showed a dramatic increase in Bax cleavage to p18 Bax compared with untreated pMOCK cells at 48 h, but this difference was not observed in pDNAJB6(S) cells in both the mitochondrial and cytosolic fractions. Choi et al. have reported that the expression levels of Bax protein are not altered during the first 40 h of MPP^+^ treatment, but that p18 Bax fragments appeared at 40 h. Our observations are consistent with the report investigating time-dependent changes in Bax and p18 Bax fragments in a DA neuronal cell line (MN9D) in the later stages of MPP^+^-induced apoptosis [28]. We reasoned, therefore, that DNAJB6(S) could be a potential repressor of p18 Bax at later stages of apoptosis. Moreover, our results showed a significant increase in the 35 kDa fragment of caspase-9 in pMOCK cells in the separated fractions after MPP^+^ treatment at 48 h, consistent with the aforementioned data [28]. In contrast, levels of this fragment were significantly lower in DNAJB6(S) cells after MPP^+^ treatment.

We also examined intracellular ROS levels to analyze whether they were affected by DNAJB6(S) expression after treatment with 500 *µ*M MPP^+^. ROS formation in MPP^+^-treated pMOCK cells significantly increased by 24 or 48 h but did not increase at any time point in MPP^+^-treated pDNAJB6(S) cells. Some observations from neuroblastoma cells treated with MPP^+^ to induce apoptosis have revealed that MPP^+^ disturbs the permeability of the mitochondrial outer membrane, leading to increases in the cytosolic levels of cytochrome c and apoptotic proteins including caspase-9 [29] as well as the generation of ROS [30].

Caspases are present as dormant proenzymes in healthy cells but are activated when cells are under stress [31]. Caspase-9 is a key activator of the caspase cascade and is important for normal brain development and apoptotic responses in thymocytes in vivo [29]. Moreover, caspase-9 knockout mice die perinatally with a markedly enlarged cerebrum [29]. Induction of apoptosis by MPP^+^ showed increases in caspase-9 protein expression in PC12 cells [32] and caspase-9 cleavage in neuroblastoma cells [33]. Our observations show that DNAJB6(S) suppressed MPP^+^-induced apoptosis not only by suppressing Bax but also by reducing events associated with apoptosis such as the loss of mitochondrial membrane potential (Δ*ψ*_*m*_), increase in intracellular ROS, and activation of caspase-9.

Because DNAJB6 is ubiquitously expressed in all human tissues and implicated limb-girdle muscular dystrophy [34], breast cancer [35], embryonic development [36], and neurodegenerative disease, it is interesting to know the interrelationships between the specific tissue and the underlying molecular mechanisms of the functional effect. In this study, the short isoform of DNAJB6 expressed dominantly in the cytoplasm was used over the long isoform of DNAJB6 expressed in the nucleus because we were interested in the cytoplasmic involvement of the DNAJB6(S) in regulating apoptosis of neuronal cells treated with MPP^+^.

Our laboratory has shown short isoform DNAJB6(S) expression in striatum in MPTP-induced mouse model of PD. The striatum in the mouse consists of striatal DA fibers, astrocytes, and about 2% of cholinergic neurons, which influence dopamine release. The astrocyte is a ubiquitous type of glial cell that is activated in response to many neurodegenerative diseases [37, 38]. According to brain sections of a random sample of Parkinsonian patients, DNAJB6 protein levels was found to be highly expressed in Parkinsonian astrocytes and localized in the center of Lewy bodies [8]. However, a recent study found that overexpression of DNAJB6(S) can itself induce neurotoxicity in primary neurons and the toxic effect is cell cycle dependent [39]. Therefore, further studies need to be performed to distinguish the different roles of DNAJB6(S) in the glial cells and neurons.

## 5. Conclusions

The present study demonstrates for the first time that the short isoform of the chaperone DNAJB6 could block cell death induced by MPP^+^. This cytoprotective behavior may be mediated by the observed changes in apoptotic signaling events such as inhibition of the increased ROS formation, Δ*ψ*_*m*_ loss, caspase-9 activation, and p18 Bax fragment cleavage.

## Supplementary Material

Supplementary Figure 1: (a) Endogenous level of DNAJB6(S) protein in various cell lines. Dnajb6(S) levels were determined in the indicated human glioblastoma lines using a DNAJB6 antibody. Band intensities were quantified by densitometer and indicated as relative fold of Dnajb6/ß-actin (b). β-Actin served as an internal control. Bands represent specific DNAJB6(S) signal (27 kDa).
Supplementary Figure 2: MPP^+^ reduces the viability of LN18 cells. (a) LN18 cells were exposed to 300 or 500 µM MPP^+^ for 24 or 48 h, and apoptosis was analyzed using flow cytometry. The total number of early (Annexin V^+^/PI^−^) and late (Annexin V^+^/PI^+^) apoptotic cells are expressed as percentages. (b)-(g) Morphological changes were examined under a phase-contrast microscope. Bar, 100 µm. (h) DNAJB6(S) protein levels were measured by western blotting, and the data are presented as the mean relative to the expression of untreated cells. β-Actin was used as an internal control. (N = 3, mean ± SEM, ∗p < 0.05 and ^n.s.^p > 0.05 compared with control at 24 or 48 h).
Supplementary Figure 3: Protein levels of DNAJB6(S) were evaluated by western blot assay after treatment with 500 µM MPP^+^ for 48 h. Results marked with dashed red lines are used in Figure 4(h). #1, #2 and #3 indicate the sample number from the separated cell culture. Beta-actin was used as an internal control. Band of red boxes were used in Figure.

## Abbreviations

DA: Dopaminergic
Hsp40: Heat shock protein 40
HSPs: Heat shock proteins
MPP^+^: 1-Methyl-4-phenylpyridinium ion
MPTP: 1-Methyl-4-phenyl-1,2,3,6-tetrahydropyridine
JC-1: 5,5′,6,6′-tetrachloro-1,1′,3,3′-tetraethylbenzimidazolylcarbocyanine iodide
PD: Parkinson's disease
p18 Bax: Truncated/cleaved form of Bax
ROS: Reactive oxygen species
Δ*ψ*_*m*_: Mitochondrial membrane potential.

## References

[1] Ritossa F. (1966). Discovery of the heat shock response. *Experientia*.

[2] Auluck P. K., Chan H. Y. E., Trojanowski J. Q., Lee V. M.-Y., Bonini N. M. (2002). Chaperone suppression of *α*-synuclein toxicity in a *Drosophila* model for Parkinson's disease. *Science*.

[3] Muchowski P. J., Wacker J. L. (2005). Modulation of neurodegeneration by molecular chaperones. *Nature Reviews Neuroscience*.

[4] Meng E., Shevde L. A., Samant R. S. (2016). Emerging roles and underlying molecular mechanisms of DNAJB6 in cancer. *Oncotarget*.

[5] Andrews J. F., Sykora L. J., Letostak T. B. (2012). Cellular stress stimulates nuclear localization signal (NLS) independent nuclear transport of MRJ. *Experimental Cell Research*.

[6] Mitra A., Fillmore R. A., Metge B. J. (2008). Large isoform of MRJ (DNAJB6) reduces malignant activity of breast cancer. *Breast Cancer Research*.

[7] Hageman J., Rujano M. A., van Waarde M. A. W. H. (2010). A DNAJB chaperone subfamily with HDAC-dependent activities suppresses toxic protein aggregation. *Molecular Cell*.

[8] Durrenberger P. F., Filiou M. D., Moran L. B. (2009). DnaJB6 is present in the core of Lewy bodies and is highly up-regulated in Parkinsonian astrocytes. *Journal of Neuroscience Research*.

[9] Chen J., Tang X. Q., Zhi J. L. (2006). Curcumin protects PC12 cells against 1-methyl-4-phenylpyridinium ion-induced apoptosis by bcl-2-mitochondria-ROS-iNOS pathway. *Apoptosis*.

[10] Wang S., He H., Chen L., Zhang W., Zhang X., Chen J. (2015). Protective effects of salidroside in the MPTP/MPP^+^-induced model of Parkinson's disease through ROS–NO-related mitochondrion pathway. *Molecular Neurobiology*.

[11] Iwashita A., Yamazaki S., Mihara K. (2004). Neuroprotective effects of a novel poly(ADP-ribose) polymerase-1 inhibitor, 2-{3-[4-(4-chlorophenyl)-1-piperazinyl] propyl}-4(3H)-quinazolinone (FR255595), in an in vitro model of cell death and in mouse 1-methyl-4-phenyl-1,2,3,6-tetrahydropyridine model of Parkinson's disease. *Journal of Pharmacology and Experimental Therapeutics*.

[12] Tatton N. A., Kish S. J. (1997). In situ detection of apoptotic nuclei in the substantia nigra compacta of 1-methyl-4-phenyl-1,2,3,6-tetrahydropyridine-treated mice using terminal deoxynucleotidyl transferase labelling and acridine orange staining. *Neuroscience*.

[13] Cassarino D. S., Parks J. K., Parker W. D., Bennett J. P. (1999). The parkinsonian neurotoxin MPP^+^ opens the mitochondrial permeability transition pore and releases cytochrome c in isolated mitochondria via an oxidative mechanism. *Biochimica et Biophysica Acta—Molecular Basis of Disease*.

[14] Guo B., Xu D., Duan H. (2014). Therapeutic effects of multifunctional tetramethylpyrazine nitrone on models of Parkinson's disease in vitro and in vivo. *Biological & Pharmaceutical Bulletin*.

[15] Tatton W. G., Chalmers-Redman R. M. E., Rideout H. J., Tatton N. A. (1999). Mitochondrial permeability in neuronal death: possible relevance to the pathogenesis of Parkinson's disease. *Parkinsonism and Related Disorders*.

[16] Brooks W. J., Jarvis M. F., Wagner G. C. (1989). Astrocytes as a primary locus for the conversion MPTP into MPP+. *Journal of Neural Transmission*.

[17] Kitayama S., Mitsuhata C., Davis S. (1998). MPP^+^ toxicity and plasma membrane dopamine transporter: study using cell lines expressing the wild-type and mutant rat dopamine transporters. *Biochimica et Biophysica Acta (BBA)—Molecular Cell Research*.

[18] Choi Y.-G., Yeo S., Hong Y.-M., Lim S. (2011). Neuroprotective changes of striatal degeneration-related gene expression by acupuncture in an MPTP mouse model of Parkinsonism: microarray analysis. *Cellular and Molecular Neurobiology*.

[19] Hengartner M. O. (2000). The biochemistry of apoptosis. *Nature*.

[20] Bredesen D. E., Rao R. V., Mehlen P. (2006). Cell death in the nervous system. *Nature*.

[21] Green D. R., Reed J. C. (1998). Mitochondria and apoptosis. *Science*.

[22] Reed J. C. (1994). Bcl-2 and the regulation of programmed cell death. *Journal of Cell Biology*.

[23] Wei M. C., Zong W.-X., Cheng E. H.-Y. (2001). Proapoptotic BAX and BAK: a requisite gateway to mitochondrial dysfunction and death. *Science*.

[24] Gross A., Jockel J., Wei M. C., Korsmeyer S. J. (1998). Enforced dimerization of BAX results in its translocation, mitochondrial dysfunction and apoptosis. *EMBO Journal*.

[25] Vermes I., Haanen C., Steffens-Nakken H., Reutellingsperger C. (1995). A novel assay for apoptosis. Flow cytometric detection of phosphatidylserine expression on early apoptotic cells using fluorescein labelled Annexin V. *Journal of Immunological Methods*.

[26] Wood D. E., Thomas A., Devi L. A. (1998). Bax cleavage is mediated by calpain during drug-induced apoptosis. *Oncogene*.

[27] Choi W.-S., Lee E.-H., Chung C.-W. (2001). Cleavage of Bax is mediated by caspase-dependent or -independent calpain activation in dopaminergic neuronal cells: protective role of Bcl-2. *Journal of Neurochemistry*.

[28] Choi W.-S., Canzoniero L. M. T., Sensi S. L. (1999). Characterization of MPP^+^-induced cell death in a dopaminergic neuronal cell line: role of macromolecule synthesis, cytosolic calcium, caspase, and Bcl-2-related proteins. *Experimental Neurology*.

[29] Kuida K., Haydar T. F., Kuan C.-Y. (1998). Reduced apoptosis and cytochrome C-mediated caspase activation in mice lacking caspase 9. *Cell*.

[30] Di Monte D., Sandy M. S., Ekström G., Smith M. T. (1986). Comparative studies on the mechanisms of paraquat and 1-methyl-4-phenylpyridine (MPP^+^) cytotoxicity. *Biochemical and Biophysical Research Communications*.

[31] Cho S.-G., Choi E.-J. (2002). Apoptotic signaling pathways: caspases and stress-activated protein kinases. *Journal of Biochemistry and Molecular Biology*.

[32] Zhou J., Sun Y., Zhao X., Deng Z., Pu X. (2013). 3-O-demethylswertipunicoside inhibits MPP^+^-induced oxidative stress and apoptosis in PC12 cells. *Brain Research*.

[33] Xu D., Duan H., Zhang Z. (2014). The novel tetramethylpyrazine bis-nitrone (TN-2) protects against MPTP/MPP^+^-induced neurotoxicity via inhibition of mitochondrial-dependent apoptosis. *Journal of Neuroimmune Pharmacology*.

[34] Sarparanta J., Jonson P. H., Golzio C. (2012). Mutations affecting the cytoplasmic functions of the co-chaperone DNAJB6 cause limb-girdle muscular dystrophy. *Nature Genetics*.

[35] Mitra A., Rostas J. W., Dyess D. L., Shevde L. A., Samant R. S. (2012). Micro-RNA-632 downregulates DNAJB6 in breast cancer. *Laboratory Investigation*.

[36] Kuo Y., Ren S., Lao U., Edgar B. A., Wang T. (2013). Suppression of polyglutamine protein toxicity by co-expression of a heat-shock protein 40 and a heat-shock protein 110. *Cell Death & Disease*.

[37] Doorn K. J., Lucassen P. J., Boddeke H. W. (2012). Emerging roles of microglial activation and non-motor symptoms in Parkinson's disease. *Progress in Neurobiology*.

[38] Teismann P., Schulz J. B. (2004). Cellular pathology of Parkinson's disease: astrocytes, microglia and inflammation. *Cell and Tissue Research*.

[39] Smith C., D'Mello S. R. (2016). Cell and context-dependent effects of the heat shock protein DNAJB6 on neuronal survival. *Molecular Neurobiology*.

