# Supplementary material for: The Short Isoform of DNAJB6 Protects against 1-Methyl-4-phenylpridinium Ion-Induced Apoptosis in LN18 Cells via Inhibiting Both ROS Formation and Mitochondrial Membrane Potential Loss
Source: Oxid Med Cell Longev. 2017 Feb 9;2017:7982389. doi: 10.1155/2017/7982389 (PMC5322441; doi:10.1155/2017/7982389)
Supplement: Supplementary file 1 — Supplementary Figure 1: (a) Endogenous level of DNAJB6(S) protein in various cell lines. Dnajb6(S) levels were determined in the indicated human glioblastoma lines using a DNAJB6 antibody. Band intensities were quantified by densitometer and indicated as relative fold of Dnajb6/ß-actin (b). β-Actin served as an internal control. Bands represent specific DNAJB6(S) signal (27 kDa). Supplementary Figure 2: MPP+ reduces the viability of LN18 cells. (a) LN18 cells were exposed to 300 or 500 µM MPP+ for 24 or 48 h, and apoptosis was analyzed using flow cytometry. The total number of early (Annexin V+/PI−) and late (Annexin V+/PI+) apoptotic cells are expressed as percentages. (b)-(g) Morphological changes were examined under a phase-contrast microscope. Bar, 100 µm. (h) DNAJB6(S) protein levels were measured by western blotting, and the data are presented as the mean relative to the expression of untreated cells. β-Actin was used as an internal control. (N = 3, mean ± SEM, ∗p < 0.05 and n.s.p > 0.05 compared with control at 24 or 48 h). Supplementary Figure 3: Protein levels of DNAJB6(S) were evaluated by western blot assay after treatment with 500 µM MPP+ for 48 h. Results marked with dashed red lines are used in Figure 4(h). #1, #2 and #3 indicate the sample number from the separated cell culture. Beta-actin was used as an internal control. Band of red boxes were used in Figure. [file 7982389.f1.pptx]

## Slide 1
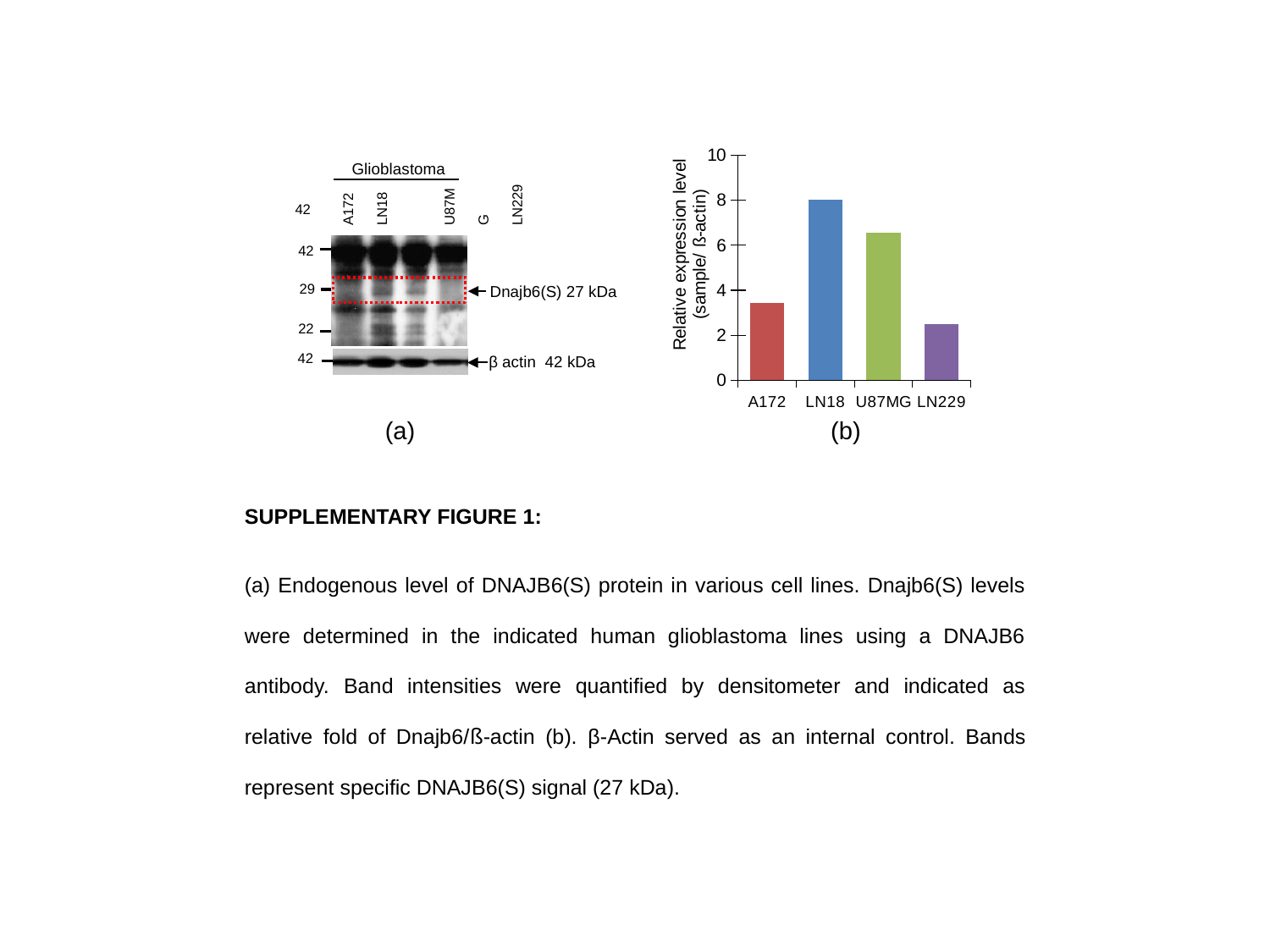

### Chart
| Category | |
|---|---|
| A172 | 3.4401223154601053 |
| LN18 | 8.028416043507818 |
| U87MG | 6.578534254756418 |
| LN229 | 2.4929270946681177 |Glioblastoma
A172
LN18
U87MG
LN229
42
42
29
Dnajb6(S) 27 kDa
22
42
β actin 42 kDa
(a)
(b)
SUPPLEMENTARY FIGURE 1:
(a) Endogenous level of DNAJB6(S) protein in various cell lines. Dnajb6(S) levels were determined in the indicated human glioblastoma lines using a DNAJB6 antibody. Band intensities were quantified by densitometer and indicated as relative fold of Dnajb6/ß-actin (b). β-Actin served as an internal control. Bands represent specific DNAJB6(S) signal (27 kDa).

## Slide 2
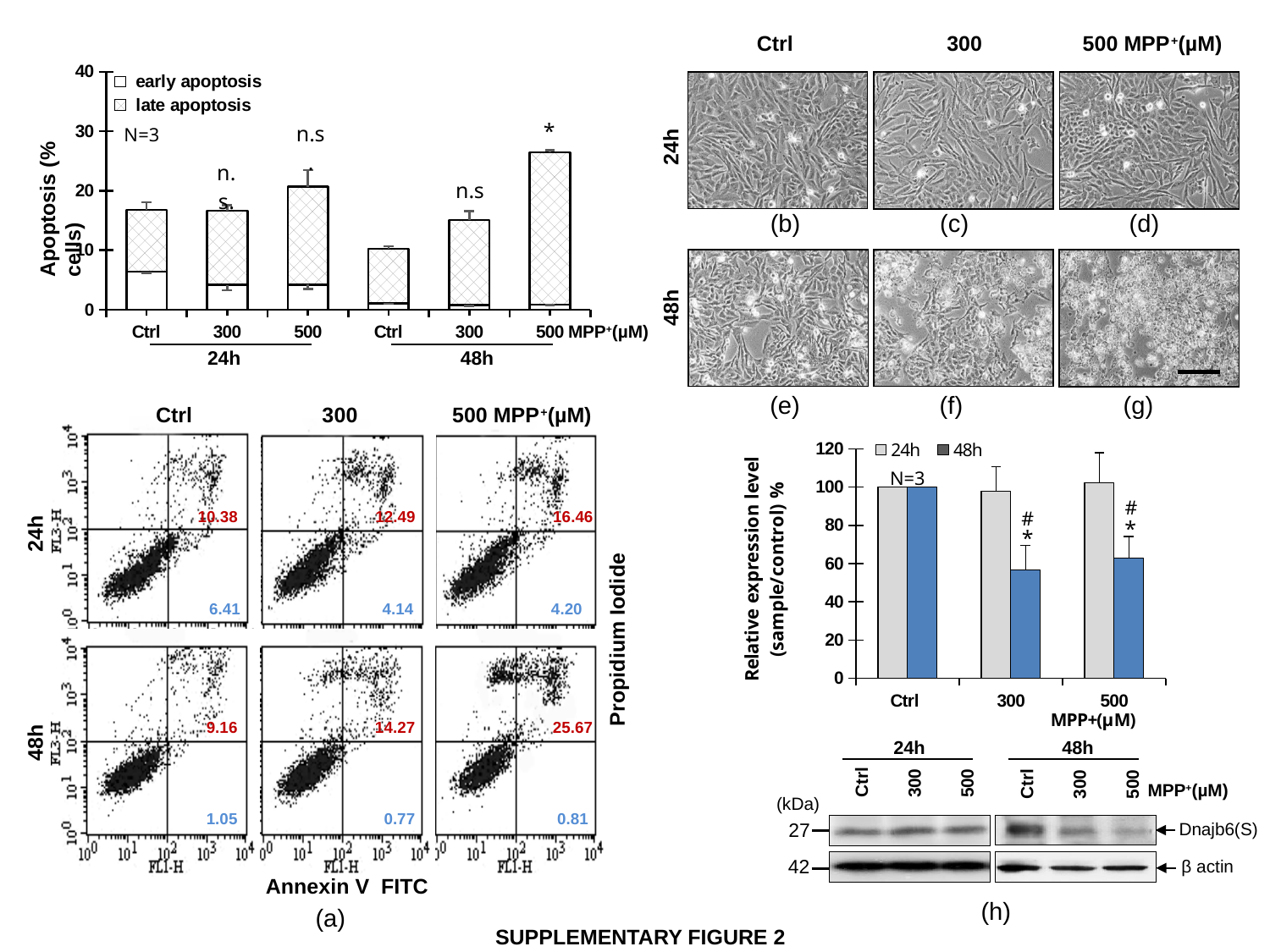

### Chart
| Category | early apoptosis | late apoptosis |
|---|---|---|
| Ctrl | 6.41 | 10.38 |
| 300 | 4.14 | 12.49 |
| 500 | 4.2 | 16.45999999999999 |
| Ctrl | 1.05 | 9.16 |
| 300 | 0.7700000000000001 | 14.27 |
| 500 | 0.81 | 25.67 |Apoptosis (% cells)
MPP+(µM)
 Ctrl 300 500 MPP+(µM)
48h 24h
(b) (c) (d)
(e) (f) (g)
24h 48h
Ctrl 300 500 MPP+(µM)
48h 24h
Propidium Iodide
Annexin V FITC
10.38 12.49 16.46
6.41 4.14 4.20
9.16 14.27 25.67
1.05 0.77 0.81
### Chart
| Category | 24h | 48h |
|---|---|---|
| Ctrl | 100.0 | 100.0 |
| 300 | 97.71927128205884 | 56.66000000000001 |
| 500 | 102.28921704503918 | 62.68 |N=3
#
#
*
*
24h 48h
Ctrl
300
500
Ctrl
300
500
MPP+(µM)
(kDa)
27
Dnajb6(S)
42
β actin
(h)
(a)
SUPPLEMENTARY FIGURE 2

## Slide 3
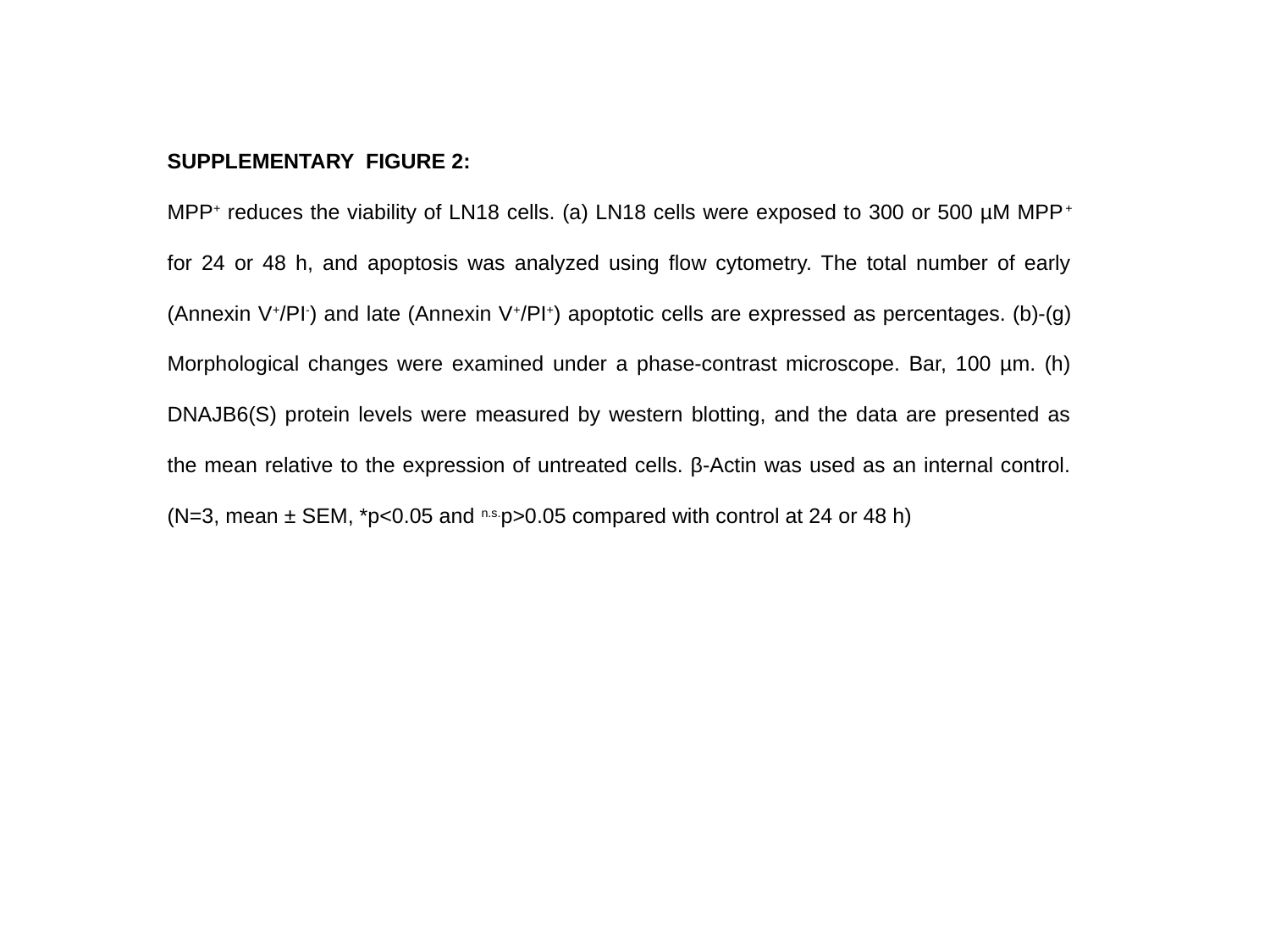

SUPPLEMENTARY FIGURE 2:
MPP+ reduces the viability of LN18 cells. (a) LN18 cells were exposed to 300 or 500 µM MPP+ for 24 or 48 h, and apoptosis was analyzed using flow cytometry. The total number of early (Annexin V+/PI-) and late (Annexin V+/PI+) apoptotic cells are expressed as percentages. (b)-(g) Morphological changes were examined under a phase-contrast microscope. Bar, 100 µm. (h) DNAJB6(S) protein levels were measured by western blotting, and the data are presented as the mean relative to the expression of untreated cells. β-Actin was used as an internal control. (N=3, mean ± SEM, *p<0.05 and n.s.p>0.05 compared with control at 24 or 48 h)

## Slide 4
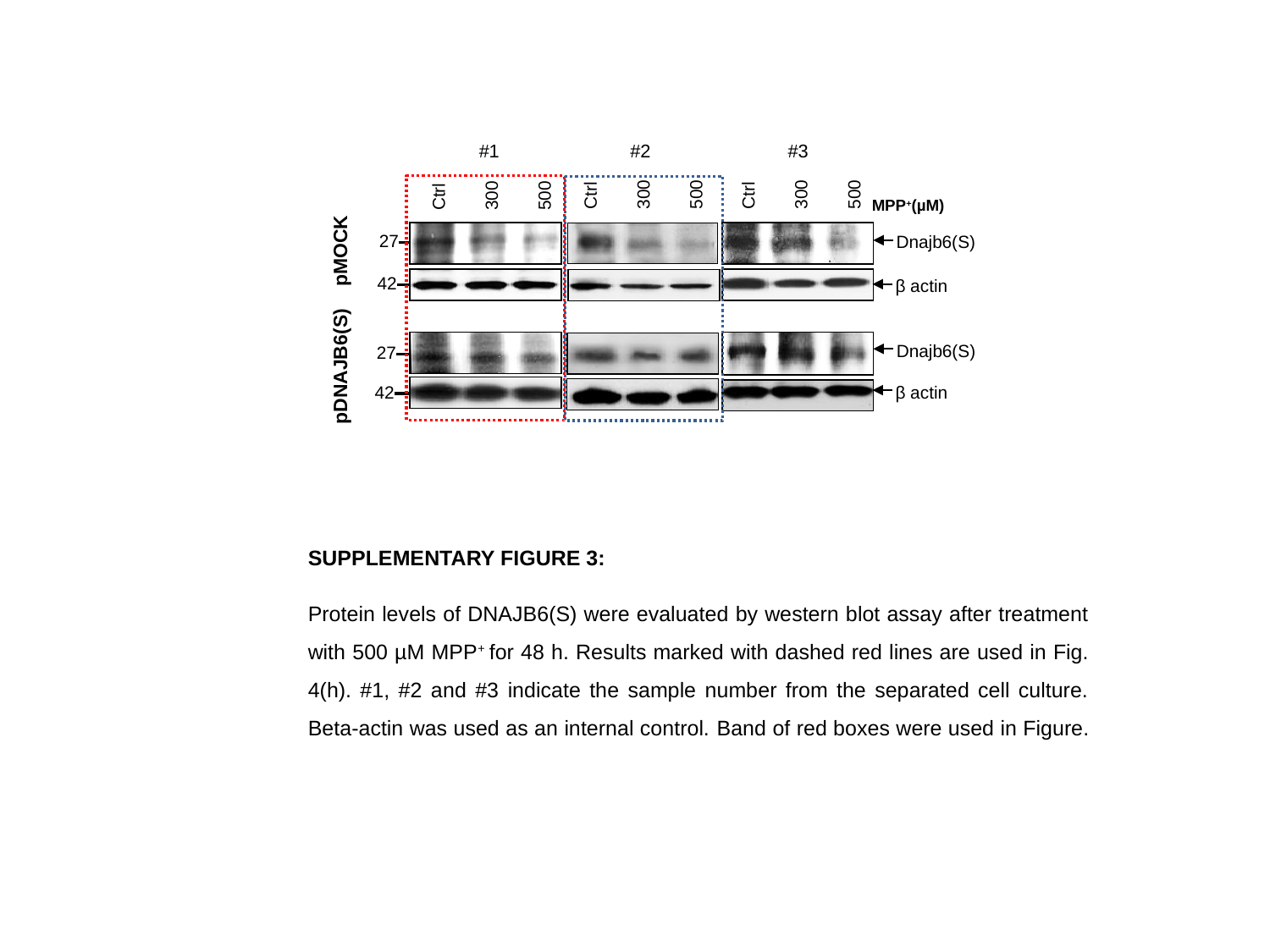

#1
#2
#3
pMOCK
Ctrl
300
500
Ctrl
300
500
Ctrl
300
500
MPP+(µM)
27
Dnajb6(S)
pDNAJB6(S)
42
β actin
Dnajb6(S)
27
42
β actin
SUPPLEMENTARY FIGURE 3:
Protein levels of DNAJB6(S) were evaluated by western blot assay after treatment with 500 µM MPP+ for 48 h. Results marked with dashed red lines are used in Fig. 4(h). #1, #2 and #3 indicate the sample number from the separated cell culture. Beta-actin was used as an internal control. Band of red boxes were used in Figure.
